# Prevalence and determinants of unintended pregnancy among women in Nairobi, Kenya

**DOI:** 10.1186/1471-2393-13-69

**Published:** 2013-03-19

**Authors:** Lawrence Ikamari, Chimaraoke Izugbara, Rhoune Ochako

**Affiliations:** 1 Population Studies and Research Institute, University of Nairobi, P.O.BOX 30197, 00100, GPO Nairobi, Kenya; 2 African Population and Research Centre (APHRC), P.O.BOX 10787, 00100 Nairobi, Kenya; 3 Population Services International (Formerly worked with APHRC), P.O.BOX 22591–00400, Nairobi, Kenya

**Keywords:** Unintended pregnancy, Determinants, Slum, Non-slum settlements, Urban, Nairobi, Kenya

## Abstract

**Background:**

The prevalence of unintended pregnancy in Kenya continues to be high. The 2003 Kenya Demographic and Health Survey (KDHS) showed that nearly 50% of unmarried women aged 15–19 and 45% of the married women reported their current pregnancies as mistimed or unwanted. The 2008–09 KDHS showed that 43% of married women in Kenya reported their current pregnancies were unintended. Unintended pregnancy is one of the most critical factors contributing to schoolgirl drop out in Kenya. Up to 13,000 Kenyan girls drop out of school every year as a result of unintended pregnancy. Unsafe pregnancy termination contributes immensely to maternal mortality which currently estimated at 488 deaths per 100 000 live births. In Kenya, the determinants of prevalence and determinants of unintended pregnancy among women in diverse social and economic situations, particularly in urban areas, are poorly understood due to lack of data. This paper addresses the prevalence and the determinants of unintended pregnancy among women in slum and non-slum settlements of Nairobi.

**Methods:**

This study used the data that was collected among a random sample of 1262 slum and non-slum women aged 15–49 years in Nairobi. The data was analyzed using simple percentages and logistic regression.

**Results:**

The study found that 24 percent of all the women had unintended pregnancy. The prevalence of unintended pregnancy was 21 per cent among women in slum settlements compared to 27 per cent among those in non-slum settlements. Marital status, employment status, ethnicity and type of settlement were significantly associated with unintended pregnancy. Logistic analysis results indicate that age, marital status and type of settlement had statistically significantly effects on unintended pregnancy. Young women aged 15–19 were significantly more likely than older women to experience unintended pregnancy. Similarly, unmarried women showed elevated risk for unintended pregnancy than ever-married women. Women in non-slum settlements were significantly more likely to experience unintended pregnancy than their counterparts in slum settlements.

The determinants of unintended pregnancy differed between women in each type of settlement. Among slum women, age, parity and marital status each had significant net effect on unintended pregnancy. But for non-slum women, it was marital status and ethnicity that had significant net effects.

**Conclusion:**

The study found a high prevalence of unintended pregnancy among the study population and indicated that young and unmarried women, irrespective of their educational attainment and household wealth status, have a higher likelihood of experiencing unintended pregnancy. Except for the results on educational attainments and household wealth, these results compared well with the results reported in the literature.

The results indicate the need for effective programs and strategies to increase access to contraceptive services and related education, information and communication among the study population, particularly among the young and unmarried women. Increased access to family planning services is key to reducing unintended pregnancy among the study population. This calls for concerted efforts by all the stakeholders to improve access to family planning services among the study population. Increased access should be accompanied with improvement in the quality of care and availability of information about effective utilization of family planning methods.

## Background

Unintended pregnancy, which includes both mistimed and unwanted pregnancies, is a global social and health challenge. Worldwide, 38% of pregnancies are unintended (that is, some 80 million pregnancies annually). In sub-Saharan Africa, unintended pregnancy accounts for more than a quarter of the 40 million pregnancies that occur annually. Unintended pregnancies increase health and economic risks for children, women, men and families. Research indicates that unintentional pregnancy is a key risk factor for adverse pregnancy and maternal outcomes, including mortality and morbidity associated with unsafe induced abortions [1-3] Unintended pregnancy has also been linked to low use of appropriate maternal health care [2,4]; [5,6]. Unintended pregnancy is also a major cause of unsafe abortion [1-3,6].

As in most of Africa, the prevalence of unintended pregnancy in Kenya continues to be high. In Adetunji's [7] study of eight sub-Saharan African countries, Kenya recorded the highest proportion of unintended childbearing. In the 2003, the Kenya Demographic and Health Survey (KDHS) showed that nearly 50% of unmarried women aged 15–19 and 45% of the married women reported their current pregnancies as mistimed or unwanted [8]. The 2008–09 KDHS showed that 43% (26% mistimed and 17% unwanted) of married women in Kenya reported their current pregnancies as unintended [9]. Unintended pregnancy is one of the most critical factors contributing to schoolgirl drop out in Kenya. Up to 13,000 Kenyan girls drop out of school every year as a result of unintended pregnancy [10] In addition, unsafe pregnancy termination contributes immensely to maternal mortality which currently estimated at 488 deaths per 100 000 live births [9].

Studies have shown a wide range of correlates of unintended pregnancy. Unintended pregnancies mostly arise as a result of nonuse or incorrect use of contraceptives, or a noticeable contraceptive failure [6,7,11]. Unintended pregnancies have also been shown to be strongly associated with maternal age and number of previous births [2,7,11-13]. A prospective study in 2 governorates of Upper Egypt revealed that the majority of women never used contraception, and unintended pregnancy was more prevalent in this category of women compared to those who had ever contraception used [14].

In Chile, women aged less than 25 and of low socioeconomic status were more likely, than their peers living in households of better socioeconomic status, to have unplanned pregnancies [15]. In Harare, a significant association was found between unintended pregnancy and age, with women aged 19 years and below or 35 years and above having a higher risk of unintended pregnancy [16]. Similar results have been reported in several other studies. Young women have higher likelihood of inconsistent or nonuse of effective family planning methods than older women and have greater risk to have mistimed than intended pregnancy [17-19]. Urban women, furthermore, are less likely than rural women to have more children than that which they regard as ideal. Research from different countries also indicate that women with better education levels were less likely than those with less education levels to have more children than that which they regard as ideal. Moreover, the higher education and the better socioeconomic status a woman had, and then it is less likely for her to have an unplanned pregnancy [2,7,11-13].

Existing literature on unintended pregnancy in Kenya has addressed its socio-demographic correlates, national prevalence, implications for maternal and child health and care-seeking, and repeatability [5,7,20]. These studies have relied largely on national large-scale or localized facility-based surveys. Little is therefore known about the prevalence and determinants of pregnancy among women from diverse socio-economic and livelihoods, particularly in urban areas of Kenya. The current study addresses the prevalence and the determinants of unintended pregnancy among women in slum and non-slum settlements of Nairobi.

Following rapid urban growth under enormous economic constraints, an increasing proportion of Kenyans now live in cities. However, urbanization in Kenya has produced critical geographic concentrations characterized by both prosperity and poverty. Cities, deeply divided along socio-economic lines, have thus emerged all over the country. Currently, high-rent neighborhoods characterized by affluence exist next to slums noted for their squalor and impoverished livelihoods. Generally, livelihood conditions vary clearly between these zones, often translating into objective differences in health outcomes [21].

Poor urban settlement contexts set limits on the ability of women and men to safeguard their sexual and reproductive health, control their fertility, and implement their fertility aspirations [21,22]. Essentially, these settlements are characterized by extreme poverty and poor livelihood conditions, limited access to family planning services, illiteracy, sexual violence, and lack of access to quality health care, including ante and post-natal care services. They present particularly interesting and fertile locations for unintended pregnancy and related behavior [21].

### Study objectives

The goal of this study was to generate new knowledge on the prevalence and determinants of unintended pregnancy among slum and non-slum women in Nairobi, Kenya. Specifically, the study sought to (a): examine the prevalence of unintended pregnancy in study settlements and (b): explore the socio economic and demographic determinants of unintended pregnancy in the study communities.

## Method

### Source of data

The data for this paper were drawn from the study on “Prevalence, Perceptions, and Experiences of Unwanted Pregnancy among women in slum and non-slum settlements of Nairobi, Kenya” conducted by the African Population and Health Research Centre (APHRC) in 2009–10. The study was conducted among women aged 15–49 years in four communities- Korogocho, Viwandani, Jericho, and Harambee in Nairobi. Korogocho and Viwandani are slum settlements whereas Jericho and Harambeeare non-slum Settlements. The study collected data from a total of 1962 randomly-selected women. A two-stage sampling design was employed to recruit study participants. The initial stage involved a random sampling of households from the settlements.

The sample of households was drawn from APHRC’s Nairobi Urban Health and Demographic Surveillance System (NUHDSS) which is implemented in these settlements. The second stage involved a simple random selection of one eligible woman in each of the sampled households. In the study, information was collected on women’s social, economic, demographic, pregnancy, birth histories (including miscarriages and or abortions, stillbirths, and neonatal deaths) as well as contraceptive behavior. It also collected information on unintended pregnancy among women, the number of times this had happened, and why the pregnancy was considered unintended. Women who admitted to experiencing unintended pregnancy were also asked how they managed the pregnancy. This paper is based on 1,272 women who re-reported ever being pregnant and who indicated whether their most recent pregnancy was intended or not.

The study was approved by the Kenya Medical Research Institute (KEMRI). Informed consent for participation was also obtained from each of the respondents.

### Study variables

The dependent variable is pregnancy intention, measured as a two-outcome variable and coded as intended pregnancy, if the pregnancy occurred at a time when the woman wanted it, and unintended pregnancy, if the pregnancy occurred at a time when the woman would have wanted it later or did not want it at all. The independent variables used in this paper include education (coded as none, primary and secondary/higher), wealth index (recoded as tertiles and labeled poor, middle and rich), ethnicity, parity, age, marital status, household size, employment status, and type of residence. These are some of the variables that have been found to affect incidence of unintended pregnancy elsewhere.

### Data analysis

The study used a mix of methods for data analysis. Simple percentages and cross-tabulation are used to analyze the levels and differentials in unintended pregnancy. Logistic regression is used in multivariate analysis of factors affecting unintended pregnancy. Results are presented as risk ratios, which represent the relative likelihood of exposure to the variable of interest. The risk ratio of the reference group or category is one (1.00). An odds ratio of greater than 1.00 indicates increased likelihood of experiencing unintended pregnancy while an odds ratio of less than 1.00 indicates a lower likelihood of experiencing unintended pregnancy. In the study, independent variables are considered significant if their effects on unintended pregnancy are statistically significant at the 95 per cent level of significance.

## Results

### Basic socio-economic and demographic characteristics of the study population

Table 1 shows the socio-demographic characteristics of 1,272 women who were ever pregnant and reported whether their last pregnancy was intended or unintended. Slightly more than half (58%) of the women were from slum settlements, majority of the women (60%), were aged 20–34 years while 43% had primary level education. More than half of the women were 62% currently married and majority of the households (54%) had between 3 and 5 persons while 59% of the women were of parity 1 and 2. Considering ethnic affiliation, Kikuyu women were the majority (34%). Twenty-four percent of the pregnancy occurring among these women was reported as unintended, meaning they occurred at a time when the woman would have preferred to have it later or did not want it at all.

**Table 1:** Percentage distribution of variables in the sample included in the analysis among women aged 15-49 years

**Characteristics**	**Frequency**	**Percent**
**Age**		
Less than 20	19	1.5
20-34	760	59.7
35-49	493	38.8
**Level of education**		
None	36	2.8
Primary	551	43.3
Secondary	438	34.4
Higher	247	19.4
**Marital status**		
Never Married	204	16.0
Currently Married	785	61.7
Formerly married	283	22.3
**Household size**		
1-2 persons	130	10.2
3-5 persons	680	53.5
6 and above	462	36.3
**Parity**		
0	20	1.6
1-2 births	744	58.5
3 and above births	508	39.9
**Wealth index**		
Poor	499	39.2
Medium	449	35.3
Rich	324	25.5
**Type of residence**		
Slum	736	57.9
Non-slum	536	42.1
**Ethnicity**		
Kikuyu	433	34.0
Luhya	228	17.9
Luo	213	16.8
Kamba	244	19.2
Other	154	12.1
**Employment status**		
Unemployed/student	484	38.1
Informal employment	152	11.9
Formal employment	234	18.4
Self employed	402	31.6
**Pregnancy intention**		
Intended pregnancy	968	76.1
Unintended pregnancy	304	23.9
**Total**	**1272**	**100.0**

### Prevalence of unintended pregnancy

About 24% of the 1272 women had unintended pregnancy. The results show statistically significant variation in the incidence of unintended pregnancy according to the number of characteristics. Never married women were more likely to experience unintended pregnancy; 62% of them reported having had unintended pregnancy compared to 13% and 26% of currently and formerly married women respectively. Women who were either unemployed or students and those in informal employment had a prevalence of 28%. Women in formal employment and those in self-employment had lower incidence of unintended pregnancy (17.5% and 22% respectively). Zero parity women had the highest prevalence of experiencing unintended pregnancy, 30% compared to 27% and 20% among those of parity 1–2 and parity 3 and above respectively. Women residing in the non-slum settlements had higher prevalence of unintended pregnancy (27%) than their counterparts in the Slum settlements (22%).

Luhya and Luo women had the highest prevalence of unintended pregnancy at 30% and 34% respectively. Women aged 15–19 had the highest prevalence of unintended pregnancy, (68%) while it was least among women aged 35–49 at 20%. Households with 1–2 persons and those with at least 6 persons experienced the highest prevalence of unintended pregnancy at 29%.

However, the results indicate no statistically significant variation in the incidence of unintended pregnancy according to the woman’s education level and the wealth index of her household (Table 2).

**Table 2:** Prevalence of unintended pregnancy among the study population, Nairobi, Kenya

	**Intended pregnancy**	**Unintended pregnancy**	**Total**
**Education**	p = 0.747		
None	83.3	16.7	100.0
Primary	76.4	23.6	100.0
Secondary	75.3	24.7	100.0
Higher	75.7	24.3	100.0
**Marital status**	p = 0.000		
Never married	38.2	61.8	100.0
Currently married	86.7	13.3	100.0
Formerly married	73.8	26.2	100.0
**Wealth index**	p = 0.969		
Poor	76.2	23.8	100.0
Medium	76.4	23.6	100.0
Rich	75.6	24.4	100.0
**Employment status**	p = 0.010		
Unemployed/student	72.5	27.5	100.0
Informal employment	71.7	28.3	100.0
Formal employment	82.5	17.5	100.0
Self employed	78.4	21.6	100.0
**Parity**	p = 0.010		
0	70.0	30.0	100.0
1-2 children	73.3	26.7	100.0
3+ children	80.5	19.5	100.0
**Residence**	p = 0.017		
Slum	78.5	21.5	100.0
Non-slum	72.8	27.2	100.0
**Ethnicity**	p = 0.000		
Kikuyu	79.5	20.5	100.0
Luhya	69.7	30.3	100.0
Luo	65.7	34.3	100.0
Kamba	80.7	19.3	100.0
Other	83.1	16.9	100.0
**Age**	p = 0.000		
15-19	31.6	68.4	100.0
20-34	74.5	25.5	100.0
35-49	80.3	19.7	100.0
**House hold size**	p = 0.000		
1-2 persons	70.8	29.2	100.0
3-5 persons	80.6	19.4	100.0
6+ persons	71.0	29.0	100.0

### Socio-economic and demographic determinants of unintended pregnancy

The results of the analysis of the determinants of unintended pregnancy among the women who took part in this study are presented in two models. Model I fitted the outcome variable and the socioeconomic variables namely: education, wealth index, employment status, ethnicity, household size and residence. Model II fitted all the variables included in Model I together with age, parity and marital status. The results of the two models are presented in Table 3.

**Table 3:** Odds ratio, based on logistic regression analysis, of unintended pregnancy among women, 15-49 in slum and non-settlements in Nairobi Kenya

**Characteristic**	**Model I**		**Model II**	
	**OR**	***p***	**OR**	***p***
**Education [Ref: None]**				
Primary	1.43	0.454	1.24	0.659
Secondary	1.32	0.562	1.11	0.830
Higher	1.52	0.417	0.99	0.982
**Wealth index [Ref: Poor]**				
Medium	0.66	0.049	0.73	0.150
Rich	0.51	0.024	0.65	0.180
**Employment status [Ref: Unemployed/student]**				
Informal employment	0.99	0.951	0.87	0.556
Formal employment	0.49	0.001	0.55	0.014
Self employed	0.72	0.045	0.80	0.237
**Ethnicity [Ref: Kikuyu]**				
Luhya	1.46	0.052	1.80	0.007
Luo	1.73	0.005	2.02	0.001
Kamba	0.91	0.664	1.18	0.460
Other	0.75	0.253	1.20	0.744
**Household size [Ref: 1-2 persons]**				
3-5 persons	0.50	0.002	0.76	0.282
6+ persons	0.74	0.205	0.97	0.922
**Residence [Ref: Slum]**				
Non-slum	2.20	0.002	1.80	0.034
**Age [Ref: 15-19]**				
20-34			0.37	0.081
35-49			0.35	0.072
**Parity [Ref: 0]**				
1-2 children			1.71	0.353
3+ children			2.04	0.235
**Marital status [Ref: Never married]**				
Currently married			0.09	0.000
Formerly married			0.21	0.000

As in the case of the bivariate analysis, the results shown in Model I indicate that education was not statistically associated with the occurrence of unintended pregnancy among the study population. However, household wealth index was closely associated with unintended pregnancy (p < 0.05). Women from medium and rich households were 66% (p < 0.05) and 51% (p < 0.02) respectively less likely to experience unintended pregnancy compared to women from poor households. Women in formal employment and those in self-employment were 49% (p < 0.001) and 72% (p < 0.05) respectively less likely to experience unintended pregnancy compared to those who were unemployed or students.

The results indicate that the likelihood of experiencing unintended pregnancy was high among Luhya and Luo women with each being 46% (p < 0.05) and 73% (p < 0.005) respectively more likely than Kikuyu women. While considering the number of persons in a household, women from households with 3–5 persons were 50% less likely to experience unintended pregnancy compared to women from households with 1–2 persons. Women in non-slum settlements were about 2.2 times (p < 0.002) more likely to experience unintended pregnancy than the women living slum settlements.

The results of Model II show that women in formal employment were 55% (p < 0.01) less likely to experience unintended pregnancy compared to those who were either unemployed or students. Luhya and Luo women also remained more likely to experience unintended pregnancy compared to their Kikuyu counterparts. Non-slum women were still 1.8 times more likely to experience unintended pregnancy compared to those from slum settlements. Women who were at least 20 years old were less likely to experience unintended pregnancy compared to those aged 15–19 years. Considering marital status, currently married and formerly married women were less likely to experience unintended pregnancy compared to those who were never married.

Further analysis of the determinants of unintended pregnancy in each of the settlements was conducted. The results for slum women are presented in Table 4 and those of non-slum women in Table 5.

**Table 4:** Odds ratio, based on logistic regression analysis, of unintended pregnancy among women, 15-49 in Slum settlements in Nairobi Kenya

**Characteristic**	**Model I**		**Model II**	
	**OR**	***p***	**OR**	***p***
**Education [Ref: None]**				
Primary	1.71	0.301	1.59	0.390
Secondary	1.74	0.295	1.54	0.441
Higher	3.42	0.106	1.50	0.640
**Wealth index [Ref: Poor]**				
Medium	1.00	0.989	1.02	0.938
Rich	0.62	0.046	0.70	0.150
**Employment status [Ref: Unemployed/student]**				
Informal employment	0.99	0.957	0.97	0.930
Formal employment	0.73	0.422	0.77	0.527
Self employed	0.92	0.713	1.00	0.998
**Ethnicity [Ref: Kikuyu]**				
Luhya	1.65	0.064	2.21	0.007
Luo	1.61	0.094	1.83	0.049
Kamba	0.61	0.073	0.76	0.360
Other	0.77	0.442	1.09	0.809
**Household size [Ref: 1-2 persons]**				
3-5 persons	0.41	0.000	0.60	0.065
6+ persons	0.77	0.331	1.06	0.862
**Age [Ref: 15-19]**				
20-34			0.20	0.030
35-49			0.19	0.031
**Parity [Ref: 0]**				
1-2 children			2.40	0.000
3+ children			2.45	0.000
**Marital status [Ref: Never married]**				
Currently married			0.12	0.000
Formerly married			0.26	0.000

**Table 5:** Odds ratio, based on logistic regression analysis, of unintended pregnancy among women, 15-49 in Non-Slum settlements in Nairobi Kenya

**Characteristic**	**Model I**		**Model II**	
	**OR**	***p***	**OR**	***p***
**Education [Ref: None]**				
Primary	0.62	0.757	0.26	0.381
Secondary	0.50	0.657	0.21	0.299
Higher	0.54	0.692	0.18	0.252
**Wealth index [Ref: Poor]**				
Medium	0.96	0.869	1.09	0.757
Rich	0.75	0.280	0.86	0.614
**Employment status [Ref: Unemployed/student]**				
Informal employment	1.10	0.803	0.91	0.844
Formal employment	0.39	0.001	0.44	0.010
Self employed	0.51	0.008	0.62	0.112
**Ethnicity [Ref: Kikuyu]**				
Luhya	1.49	0.168	1.84	0.069
Luo	2.12	0.009	2.54	0.006
Kamba	1.90	0.050	3.31	0.002
Other	0.80	0.590	1.32	0.553
**Household size [Ref: 1-2 persons]**				
3-5 persons	0.92	0.882	1.45	0.569
6+ persons	1.03	0.949	1.25	0.732
**Age [Ref: 15-19]**				
20-34			0.86	0.898
35-49			0.85	0.890
**Parity [Ref: 0]**				
1-2 children			0.62	0.536
3+ children			0.95	0.945
**Marital status [Ref: Never married]**				
Currently married			0.06	0.000
Formerly married			0.15	0.000

The results show that in both types of settlements, ethnicity and marital status have each statistically significant effect on unintended pregnancy. In both settlements, single women were significantly more likely to experience unintended pregnancy than their currently married or formerly married counterparts. Similarly, in both settlements, being Luo or Luhya was associated with a higher likelihood of experiencing unintended pregnancy compared to being a Kikuyu. Parity and age are the other factors that have statistically significant effects in the slum settlements only. In the slum settlements, the likelihood of experiencing unintended pregnancy increased with parity. For example, women of parity 1–2 children and those of at least parity 3 were 2.4 and 2.5 times, respectively, as likely as women of zero parity to experience unintended pregnancy. In contrast, the likelihood of experiencing unintended pregnancy among the women in the slum settlements declined with the age of the woman. Young women (15–19) are significantly more likely to experience unintended pregnancy compared to older women (20–34 or 35–49).

## Discussion

This study addressed the prevalence, socio-economic and demographic correlates of unintended pregnancy among slum and non-slum women in Nairobi. About 24% of the women reported an unintended pregnancy. The 2008–09 KDHS indicates that 43% of Kenyan women and 29.4% of women in Nairobi Province in particular reported their most recent pregnancies to be unintended. Judging by these figures, the incidence of unintended pregnancy among women in the study was slightly lower than the national and provincial averages. While many factors can explain the comparatively lower incidence of unintended pregnancy in the study communities, the growing availability of family planning products in the study communities needs to be highlighted. The study areas have also been the targets of aggressive family planning campaigns in recent times [23].

In the study, education and household wealth were surprisingly not strongly associated with unintended pregnancy. This is contrary to many other studies on the correlates of unintended pregnancy [24,25]. However, judging by results of the logistic regression, it is difficult to entirely dismiss the influence of income and educational levels on unintended pregnancies among the women we studied. This is because employment status was a strong predictor of unintended pregnancy. Women in formal employment were also 55% (p < 0.01) less likely to experience unintended pregnancy compared to unemployed women or students. Formal employment is related in critically important ways to earning capacity, educational levels, and social networks [26-29]. Women in formal employment tend to earn more, have higher levels of reproductive health knowledge and participate in social networks that support family planning and reduced fertility [29-34].

Further, similar to previous work from Kenya, we found that young and unmarried women in the study were, irrespective of their educational attainment and household wealth status, at high risk of experiencing unintended pregnancy [21,22,35,36]. Research in Kenya generally, and in the specific areas that we covered indicates that young people have limited access to quality sexual and reproductive health information, including knowledge on contraception [5,6,19,21,22,36]. In addition to the unaffordability of family planning products and services to many girls, several hospitals and clinics in Kenya where family products and services – mainly, oral contraceptives, injectables, implants, male condoms, IUDs and, very rarely, vasectomy – are subsidized or provided free of charge regularly experience stock outs and a dearth of qualified providers [37]. Stigma, inadequate sexuality information and cultural pressure to appear sexually chaste and inexperienced also hinder utilization of family planning services among young girls and unmarried girls [38]. Most young girls in Kenya also rely on parents, equally uninformed peers, and popular media for information on sexual maturation, pregnancy and sexuality, who often do not provide them correct and timely information, exposing them to sexual and reproductive health risks including unintended pregnancies [21,36,39,40].

Interestingly too, ethnicity had statistically significant effect on unintended pregnancy. In both settlements, being Luo or Luhya was associated with a higher likelihood of experienced unintended pregnancy compared to being a Kikuyu. Due largely to their growing access to higher education, greater participation in paid employment and widespread use of contraceptives, the Kikuyu as a whole has continued to experience steadydeclines in fertility in the recent past. On the other hand, while there is evidence of higher levels of pronatalism among the Luyha and Luo than among the Kikuyu, there is also research suggesting that women from the former ethnic groups are among the poorest and least empowered in Kenya [41-44]. The rates of unintended pregnancies from the Provinces where the Luo and Luyha traditional reside, e.g., Western and Nyanza Provinces are also among the highest in Kenya [5,19]. Higher levels of unintended pregnancy among the Luo and Luyha in the study may relate to their lower empowerment status, poorer socio-economic status and more limited access to family planning products [21,45,46]. Compared to Kikuyu women, more Luo and Luhya women in Kenya currently survive as dependents of men in relationships that hinder their power to protect themselves from unintended pregnancies and sexually transmitted infections [21,47,48]. The poorer socio-cultural and economic status of Luo and Luyha women finds expression, among other things, in the ideology of patrilineal inheritance, male-biased property rights, virilocalism and male ownership of children. For instance, among the Luo, upon the death of a husband, his widow(s) is also eventually claimed as property by brothers-in-laws or other male relatives of the deceased husband [21]. On the other hand, in the Kikuyu culture, children and the responsibility of catering for them are primarily perceived as women’s, not men’s. Kikuyu women may thus be much keener in looking for ways to avoid pregnancies that they are not well-prepared for [49,50].

## Conclusion

The study found a high prevalence of unintended pregnancy among the study population. Young and unmarried women, irrespective of their educational attainment and household wealth status, had a higher likelihood of experiencing unintended pregnancy. Except for the results on educational attainments and household wealth, our findings compared with the results reported in the literature.

The study results are critical and raise the need for programs and strategies to improve access to contraceptive services and related education, socio-economic status and information and communication particularly among the young and unmarried women. Increased availability and accessibility of family planning services is crucial to reducing unintended pregnancy among the study population. Concerted and coordinated multi-sectoral efforts are key to expanding access and uptake of family planning services. Increased access should be accompanied with improvement in the quality of care and availability of information about effective utilization of family planning methods.

## Competing interests

The authors have no competing interests.

## Authors’ contribution

LI: Conceptualized the paper and led the literature review. CI: Conceived and led the study project and wrote sections of the manuscript. RO: conducted data analysis, wrote the results section and reviewed drafts of the manuscript. All authors read and approved the final manuscript.

## Pre-publication history

The pre-publication history for this paper can be accessed here:

http://www.biomedcentral.com/1471-2393/13/69/prepub
